# Decoding Digital Visual Stimulation From Neural Manifold With Fuzzy Leaning on Cortical Oscillatory Dynamics

**DOI:** 10.3389/fncom.2022.852281

**Published:** 2022-03-11

**Authors:** Haitao Yu, Quanfa Zhao, Shanshan Li, Kai Li, Chen Liu, Jiang Wang

**Affiliations:** School of Electrical and Information Engineering, Tianjin University, Tianjin, China

**Keywords:** neural manifold, visual stimulation, brain dynamics, decoding, machine learning

## Abstract

A crucial point in neuroscience is how to correctly decode cognitive information from brain dynamics for motion control and neural rehabilitation. However, due to the instability and high dimensions of electroencephalogram (EEG) recordings, it is difficult to directly obtain information from original data. Thus, in this work, we design visual experiments and propose a novel decoding method based on the neural manifold of cortical activity to find critical visual information. First, we studied four major frequency bands divided from EEG and found that the responses of the EEG alpha band (8–15 Hz) in the frontal and occipital lobes to visual stimuli occupy a prominent place. Besides, the essential features of EEG data in the alpha band are further mined *via* two manifold learning methods. We connect temporally consecutive brain states in the *t* distribution random adjacency embedded (t-SNE) map on the trial-by-trial level and find the brain state dynamics to form a cyclic manifold, with the different tasks forming distinct loops. Meanwhile, it is proved that the latent factors of brain activities estimated by t-SNE can be used for more accurate decoding and the stable neural manifold is found. Taking the latent factors of the manifold as independent inputs, a fuzzy system-based Takagi-Sugeno-Kang model is established and further trained to identify visual EEG signals. The combination of t-SNE and fuzzy learning can highly improve the accuracy of visual cognitive decoding to 81.98%. Moreover, by optimizing the features, it is found that the combination of the frontal lobe, the parietal lobe, and the occipital lobe is the most effective factor for visual decoding with 83.05% accuracy. This work provides a potential tool for decoding visual EEG signals with the help of low-dimensional manifold dynamics, especially contributing to the brain–computer interface (BCI) control, brain function research, and neural rehabilitation.

## Introduction

The human brain readily makes sense of visual images with specific dynamics in a complex environment, but how to quantify the visual response remains poorly understood (Kourtzi and Kanwisher, 2000; Pasley et al., 2012). In the past decade, the anatomy of visual conduction in the brain is well known: the visual information is transmitted through neural pathways from retina to cortex, which triggers specific dynamics to achieve cognitive functions such as memory and envision (de Beeck et al., 2008; Wen et al., 2018). Accordingly, decoding human brain activity triggered by visual stimuli has a significant impact on brain–computer interface (BCI), brain-inspired computing, and machine vision research (Hogendoorn and Burkitt, 2018). Although it has been demonstrated that human brain activity can be decoded from neurological data in recent research (Zheng et al., 2020), with the neurological data tending to be high dimensional and unstable, it is difficult to decode useful information directly from the complex neural data. How to decode visual information from brain activity remains a tantalizingly unsolved problem in neuroscience.

The research of specific links among brain activity, cognitive behavior, and decoding information from the brain has gained increasing attention. Early studies focus on the level of a single neuron. Recently, Cunningham and Byron point out that the majority of sensory, cognitive, and motor functions depend on the interactions among many neurons, and data cannot be fundamentally understood based on a single neuron (Cunningham and Byron, 2014). Consequently, this study of the neural system is undergoing a transition from a single neuron level to a population level (Pandarinath et al., 2018). With the development of electrophysiology and neuroimaging techniques, it has been acknowledged that neural population activities collected through neurophysiology [electroencephalogram (EEG)/magnetoencephalography (MEG)] and neuroimaging techniques [e.g., functional magnetic resonance imaging (fMRI)] are influenced by external stimulus about the categories of the visual object (Spampinato et al., 2017). Neural recordings with high temporal resolution are now readily obtained *via* EEG technique (Nunez and Srinivasan, 2006; Müller et al., 2008; Schirrmeister et al., 2017). However, the brain activity recordings pose severe decoding problems because of their time-varying spectral components, highly non-stationary properties and the multiple unknown noise. Alternatively, the activity recordings of the brain have been mostly analyzed by time-frequency analysis, complex network, and so on, which extract activity features from one side and make it hard to decode directly (Yu et al., 2019a). Thus, a new analytical method is needed to decode brain activity directly from a population perspective and investigate the visual mechanisms.

Recent advances in neuroscience have demonstrated that a neural manifold is present across the brain, which provides an idea for decoding brain activity. Manifold is the subregion that can capture behavior in a given task. Due to the high degree of correlation and redundancy across individual neural activity, the dimension of the neural system is less than the number of neurons (Levina and Bickel, 2005). Complex population activity can be explained by fewer unobservable latent factors and the latent factors change over time to form a manifold. Seung and Lee (2000) have proved that there is a stable balance state in brain cognitive activities through experiments and proposed that manifold learning might be a natural behavior mode in human cognition. Degenhart et al. (2020) demonstrated that it was possible to solve several neural recording instabilities such as baseline shifts, unit dropout, and tuning changes leveraging the low-dimensional structure present in neural activity. The existence of manifolds in the brain provides a novel idea and effective tool to decode the neural population activity using the dimensionality reduction technique.

Dimensionality reduction techniques allow us to investigate neural population dynamics by drawing the neural manifold and identifying relevant population features. Several explanatory variables can be discovered and extracted from the high-dimensional data according to a specific objective of different dimensionality reduction methods. Due to these explanatory variables being not directly observed, they are often referred to as latent factors (Gallego et al., 2017). Juan et al. have confirmed that brain function is based on the latent factors rather than on the activity of a single neuron (Gallego et al., 2017). They thought that latent factors are the elemental units of volitional control and neural computation in the brain (Gallego et al., 2018). In addition, the time processes of neural responses may vary substantially in experiments on the same task, especially in cognitive tasks such as attention and decision-making. In this case, averaging responses across trials tend to obscure the neural time course of interest, and the single-trial analyses are essential. Therefore, it is necessary to extract the latent factors of the neural population activity from a single-trial level.

A proven idea of data dimensionality reduction is manifold learning, which can obtain the eigenstructure information from the data that is consistent with human cognition. The common dimensionality reduction techniques can be divided into two categories (Wu and Yan, 2009). One is the linear method used for extracting linear manifolds, whose mapping function is linear and usually has explicit expression forms, such as principal component analysis (PCA) and multidimensional scaling (MDS) (Seung and Lee, 2000; Flint et al., 2020). The other is the non-linear method used for extracting the manifolds with non-linear structural characteristics, whose mapping function is a non-linear function, always without an explicit expression. The non-linear methods present the low-dimensional embedding representation of original high-dimensional data utilizing the implicit mapping, such as local linear mapping (LLE) and *t*-distribution random adjacency embedded (t-SNE) (Lin et al., 2020). Although these methods have deep similarities, the choice of method can have a significant bearing on the scientific interpretations of brain research.

Based on these analyses, this study aims to investigate visual decoding with the neural manifold in cortical activity found by PCA or t-SNE from a single-trial level. EEG signals are directly recorded from the human cortical surface in both resting and several tasks. Moreover, we aim to research the performance of decoding visual information by comparing the two types of manifold learning methods using one of the four types of decoders, that is, Takagi-Sugeno-Kang (TSK), linear Kalman filter (LKF), long–short-term memory-recurrent neural network (LSTM-RNN), and naïve Bayes (NB). Additionally, the different performances among brain regions are examined. In this work, the decoding methods are applied to the multichannel EEG signals in the cortical of healthy people that perform a visual cognitive task. The combination of t-SNE and fuzzy learning and the feature is extracted from the frontal lobe, the parietal lobe, and the occipital lobe which can achieve the highest performance with an accuracy of 83.05%.

## Experiments and Methods

### Datasets

#### Experiment Design

**Figure 1:**
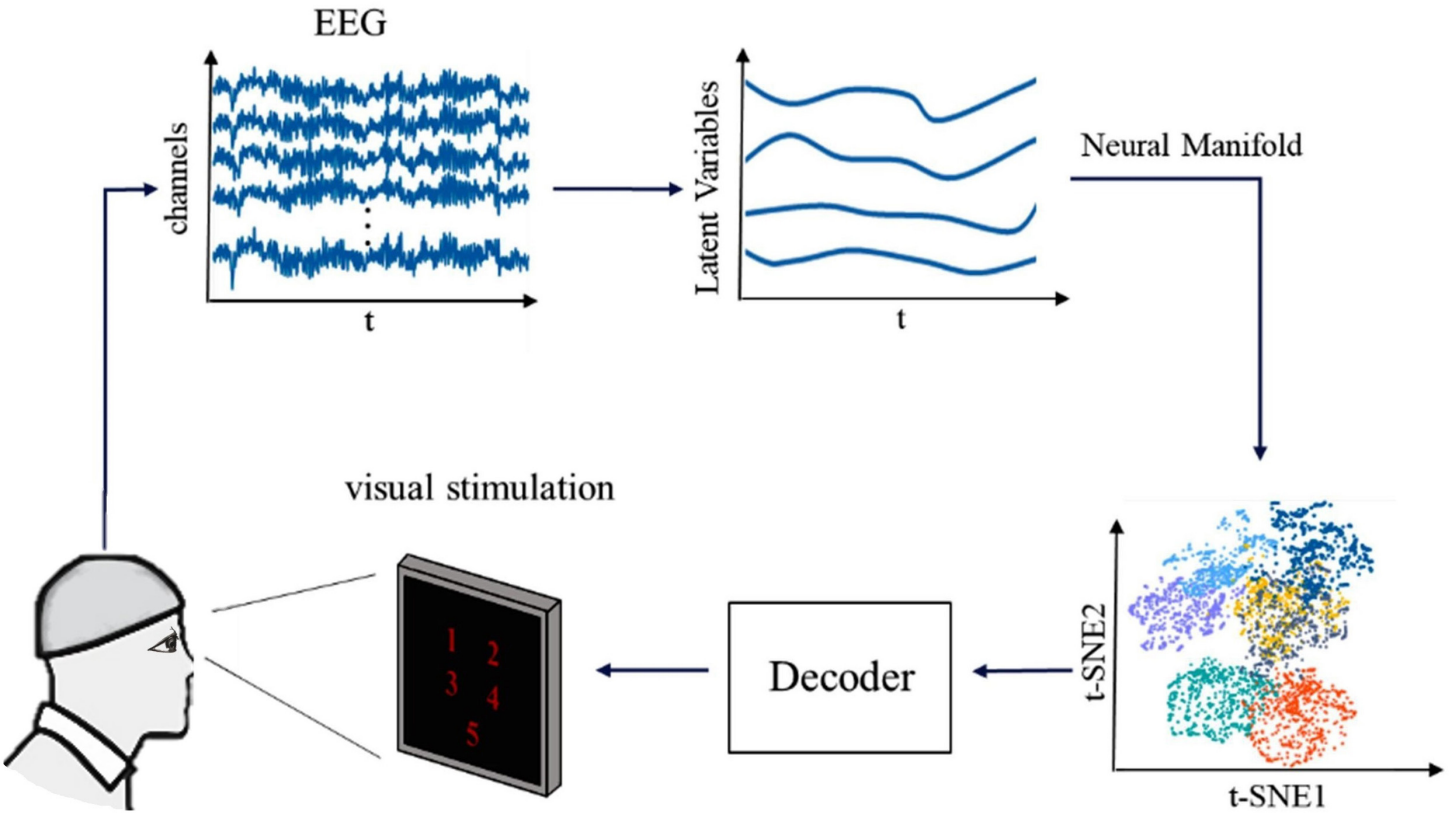
The framework of our research for decoding the visual information from brain activities. First multichannel EEG signals of five visual stimuli of volunteers are acquired and data preprocessing analysis is performed. Second, the latent factors are extracted, and the neural manifold dynamics are obtained. Finally, we combined various decoders to decode visual information.

We designed an experiment to study decoding the visual information from brain activities (Figure 1). Ten volunteers (five women and five men) between the ages of 22 and 25 participate in the experiment. All of the volunteers signed informed consent forms, and all the protocols were conformed to the guidelines contained within the Declaration of Helsinki. All participants have no neurological, psychological disorders, or any other ingredients that might influence EEG activity and agree to coordinate the experiment verbally. In each experiment, the participant's brain activity is recorded by an EEG amplifier (NIHON KOHDEN) from the 64 Ag/AgCl scalp electrodes. The sampling rate of the EEG device is 1,000 Hz, and the rate of the hardware filter is 0.5–70Hz. Each participant has been informed of the steps of the experiment before the experiment begins. The participants sit in a comfortable chair about 0.5 m in front of a 22-inch visual display. Trials begin with a blank display for 120 s, during which the participants rest. Then, the numbers from 1 to 5 are presented on the screen for viewing for 30 s, followed by a 60-s rest (blank display). The five numbers from 1 to 5 are presented randomly to prevent people's conventional wisdom from influencing the results. Participants are asked to focus on what they see during the viewing and try to recognize the number. During the experiment, the participants sit calmly in a darkened room and are asked to stare at the screen as still as possible.

#### Data Preprocessing

Raw EEG data are contaminated by artifacts from many non-physiological (power line, bad electrode contact, broken electrodes, etc.) and physiological (cardiac pulse, muscle activity, sweating, movement, etc.) sources. These artifacts have to be carefully identified and either removed or excluded from further analysis (Michel and Brunet, 2019). We used a Butterworth filter to realize temporal filtering, of which the bandwidth was 0.1–50 Hz. Then, the subsequent independent component analysis method has been applied to detect and correct artifacts. Finally, the data were visually inspected, and those bad epochs influenced by transient artifacts were rejected.

#### Divide Subbands

To investigate the changes in brain dynamics during different tasks, 30-s length of EEG signals at the previsual stimuli, visual stimuli, and postvisual stimuli states are selected. The previous study shows that the changes in EEG during visual stimuli might occur between 0 and 30 Hz (Adebimpe et al., 2016). So, we studied the four main subbands of EEG: delta (1–4 Hz), theta (4–8 Hz), alpha (8–15 Hz), and beta (15–30 Hz), which are thought to be associated with cognitive activity and typically used in synchronization analysis (Müller et al., 2008; Adebimpe et al., 2016; Kobak et al., 2016; Shin et al., 2020). Therefore, a band-passed finite impulse digital filter based on fast Fourier transform is adopted to decompose the EEG data of each channel into four subbands. All analyses in this paper are performed in MATLAB 2017b.

### Power Spectrum Density

The autoregressive (AR) Burg approach is applied to estimate the absolute power spectrum density (PSD) for each channel (Faust et al., 2008). To reach the objective of power comparisons in four frequency bands and different experimental states, the EEG data are segmented into 1-s epochs with 0.5-s overlap. A power spectrum analysis is performed for each time window. At a certain subband, the final power value at a certain time is obtained by averaging all power values of the frequency band on the current time window interval. The theoretical basis of the AR method is that the given signal is the output of a linear system whose input is white noise, which is

(1) $$\begin{array}{l} r\left(n\right)=-\overset{k}{\underset{i=1}{\sum }}a_{i}r\left(n-i\right)+u\left(n\right) \end{array}$$

where *a*_*i*_ represents the AR coefficients, *k* is the order of the AR model, and *u*(*n*) is the white noise with a variance of σ^2^. The order *k* can be described by AR parameters $\left\{a_{1},a_{2},\ldots ,a_{n},\sigma^{2}\right\}$. The PSD is defined as

(2) $$\begin{array}{l} {\hat{P}}_{AR}\left(f\right)=\;\frac{\sigma^{2}}{|1+\sum_{1}^{p}a_{i}e^{-j2\pi fi}{|}^{2}} \end{array}$$

### Principal Component Analysis

Principal component analysis is the common linear dimensionality reduction technique (Duffy et al., 1992). In this research, PCA is applied to the EEG of all subjects. To avert the case where channels with excessive fluctuations impact decoding, the EEG data are smoothed through a Gaussian kernel before employing dimensionality reduction techniques. In this research, PCA is performed to the EEG data of a single-trial level rather than trial averaged from all subjects to avoid the inaccuracy caused by averaging the responses across entire trials. The purpose of PCA is to extract principal components (PCs) from smoothed EEG data. Meanwhile, all PCs are arranged in descending order according to the magnitude of explained variances. The first 2 PCs are selected to decode visual stimuli.

### t-Distributed Stochastic Neighbor Embedding

Compared with PCA, t-SNE is a non-linear dimensionality reduction technique. t-SNE calculates the probability similarity for points using a normalized Gaussian kernel in a high-dimensional space (Van der Maaten and Hinton, 2008). Correspondingly, in low-dimensional space, the similarity is calculated through t-distribution. The similarity between sample points in high-dimensional space can be expressed by the asymmetric point probability distribution *p*_*ij*_. For the t-SNE algorithm, the *p*_*ij*_ is defined as follows:

(3) $$\begin{array}{l} p_{ij}=\frac{exp\left(\frac{-||r_{i}-r_{j}|{|}^{2}}{2{\sigma_{i}}^{2}}\right)}{\sum_{k}\sum_{l\neq k}exp\left(\frac{-||r_{k}-r_{l}|{|}^{2}}{2{\sigma_{i}}^{2}}\right)},\forall i,j\text{ and }i\neq j \end{array}$$

(4) $$\begin{array}{l} p_{ij}=\frac{p_{j|i}+p_{i|j}}{2N} \end{array}$$

where σ_*i*_ is the variance of a Gaussian centered on *r*_*i*_. Due to the different distribution density of sample points in the dataset, σ_*i*_ corresponding to different sample points is also different. The denser the distribution of data point is, the smaller σ_*i*_ is. The sparser the distribution of data point is, the larger σ_*i*_ is. The value of σ_*i*_ is calculated by binary search.

### Takagi-Sugeno-Kang Fuzzy System

Takagi-Sugeno-Kang is a fuzzy model (Takagi and Sugeno, 1985). For an input dataset $U=\left\{u_{1},u_{2},\ldots ,u_{n}\right\}\in R^{d}$ and the corresponding class label *O* = {*o*_1_, *o*_2_, …, *o*_*n*_} ($o_{i}={\left[o_{i,1},o_{i,2},\ldots o_{i,j}\right]}^{T}$). When the *i*th sample data belong to *j*th class, *o*_*i,j*_ = 1; Otherwise, *o*_*i,j*_ = 0. The *k*th fuzzy inference rules are usually described as

(5) $$\begin{array}{r} R^{k}:IF\;u_{1}\text{ is }A_{1}^{k}\wedge u_{2}\text{ is }A_{2}^{k}\wedge \ldots \wedge u_{d}\text{ is }A_{d}^{k},\\ \;THEN\;f_{k}\left(u\right)=\beta_{0}^{k}+\beta_{1}^{k}u_{1}+\ldots +\beta_{d}^{k}u_{d},k=1,\ldots ,K \end{array}$$

where the input vector of each fuzzy rule is represented as $u={\left[u_{1},u_{2},\ldots u_{d}\right]}^{T}$, $A_{i}^{k}$ are Gaussian antecedent fuzzy sets subscribed by the input variable *u*_*i*_ of Rule *k*, the number of fuzzy rules is *K*, ∧ is a fuzzy conjunction operator, the *k*th fuzzy inference rule *f*_*k*_(*u*) can be expressed by a linear combination of the input vector $u={\left[u_{1},u_{2},\ldots u_{d}\right]}^{T}$, and $\beta_{i}^{k}$ are linear coefficients.

If the input vector of each rule is *u*, the output of TSK is calculated as

(6) $$\begin{array}{l} õ=\overset{K}{\underset{k=1}{\sum }}\frac{\mu_{k}\left(u\right)f_{k}\left(u\right)}{\sum_{k^{{\;}^{\prime }}}^{K}\mu_{k^{{\;}^{\prime }}}\left(u\right)}=\overset{K}{\underset{k=1}{\sum }}{\tilde{\mu }}_{k}\left(u\right)f_{k}\left(u\right) \end{array}$$

where $\mu_{k}\left(u\right)=\prod_{i=1}^{d}\mu_{A_{i}^{k}}\left(u_{i}\right)$ is the fuzzy membership function and the normalized fuzzy membership function of the *k*th fuzzy rule is denoted as ${\tilde{\mu }}_{k}\left(u\right)=\frac{\mu_{k}\left(u\right)}{\overset{K}{\underset{k^{{\;}^{\prime }}}{\sum }}\mu_{k^{\prime }}\left(u\right)}$. $\mu_{A_{i}^{k}}\left(u_{i}\right)$ is Gaussian membership function for fuzzy set $A_{i}^{k}$, which is defined as

(7) $$\begin{array}{l} \mu_{A_{i}^{k}}\left(u_{i}\right)=exp\left[-\frac{{\left(u_{i}-c_{i}^{k}\right)}^{2}}{\delta_{i}^{k}}\right] \end{array}$$

where the element $c_{i}^{k}$ is *k*th cluster center parameters that can be obtained through the classical fuzzy c-means (FCM) (Bezdek et al., 1984) clustering algorithm:

(8) $$\begin{array}{l} c_{i}^{k}=\frac{\sum_{j=1}^{N}x_{jk}u_{ji}}{\sum_{j=1}^{N}x_{jk}} \end{array}$$

where the width parameter $\delta_{i}^{k}$ can be estimated by:

(9) $$\begin{array}{l} \delta_{i}^{k}=\frac{l\cdot \sum_{j=1}^{N}u_{jk}{\left(x_{jk}-c_{i}^{k}\right)}^{2}}{\sum_{j=1}^{N}u_{jk}} \end{array}$$

where *x*_*jk*_ ∈ [0, 1] is the fuzzy membership between *j*th input vector and *k*th cluster (*k* = 1, 2, …, *k*), and the constant *l* is the scale parameter.

Given *u*_*n*_ as the input sample, we can obtain

(10) $$\begin{array}{l} u_{n,e}={\left(1,{u_{n}}^{T}\right)}^{T} \end{array}$$

(11) $$\begin{array}{l} {ũ}_{n}^{k}={\tilde{\mu }}^{k}\left(u_{n}\right)u_{e} \end{array}$$

(12) $$\begin{array}{l} \rho \left(u_{n}\right)=\left[{\left({ũ}_{n}^{1}\right)}^{T},{\left({ũ}_{n}^{2}\right)}^{T},\ldots ,{\left({ũ}_{n}^{K}\right)}^{T}\right]\in R^{K\left(d+1\right)} \end{array}$$

(13) $$\begin{array}{l} \beta^{k}={\left(\beta_{0}^{k},\beta_{1}^{k},\ldots ,\beta_{d}^{k},\right)}^{T} \end{array}$$

(14) $$\begin{array}{l} \beta_{g}=\left[{\left(\beta^{1}\right)}^{T},{\left(\beta^{2}\right)}^{T},\ldots ,{\left(\beta^{K}\right)}^{T}\right] \end{array}$$

Thereby, the output result õ_*n*_ of a TSK fuzzy decoder for the input vector *u*_*n*_ can be described as

(15) $$\begin{array}{l} {õ}_{n}={\beta_{g}}^{T}\rho \left(u_{n}\right) \end{array}$$

### Naïve Bayes

The NB decoder is a type of Bayesian decoder that determines the probabilities of different outcomes, which can predict the most probable outcome. For the input dataset $U=\left\{u_{1},u_{2},\ldots ,u_{n}\right\}\in R^{d}$ with *m* classes and a sample *C* = {*c*_1_,*c*_2_, …, *c*_*n*_}, Bayes' theorem can be defined as

(16) $$\begin{array}{l} P\left(u_{i}|C\right)=\frac{P\left(C|u_{i}\right)P\left(c_{i}\right)}{P\left(C\right)} \end{array}$$

Because *P*(*C*) is constant for all classes, the probability that *C* belongs to each class can be estimated by

(17) $$\begin{array}{l} P\left(u_{i}|C\right)\propto P\left(c_{i}\right)\overset{n}{\underset{k=1}{\prod }}P\left(c_{k}|u_{i}\right)=\frac{\prod_{k=1}^{n}P\left(c_{k}|u_{i}\right)}{{\left[P\left(u_{i}\right)\right]}^{n-1}} \end{array}$$

According to this formula, the class with the highest probability is taken as the class of *C*.

### Long Short-Term Memory in Recurrent Neural Network

An LSTM-RNN based on an RNN architecture has been well befitting in decoding brain activities (Tsiouris et al., 2018). The LSTM-RNN is constituted by memory cell *c*, forget gate *f*, input gate *i*, and output gate *o*. LSTM-RNN can accommodate the information flow through a cell *via* these gates, and each gate can be expressed by

(18) $$\begin{array}{l} f_{t}=\sigma_{sigmoid}\left(W_{f,z}u_{t}+W_{f,1}h_{t-1}+b_{f}\right) \end{array}$$

(19) $$\begin{array}{l} i_{t}=\sigma_{sigmoid}\left(W_{i,z}u_{t}+W_{i,1}h_{t-1}+b_{i}\right) \end{array}$$

(20) $$\begin{array}{l} o_{t}=\sigma_{sigmoid}\left(W_{o,z}u_{t}+W_{o,1}h_{t-1}+b_{o}\right) \end{array}$$

where *b* is the vector of biases, *h* denotes the vector of the hidden layer, and *W* represents the weight matrix of recurrent connection from the input gate to the output gate. The input vector *u*_*t*_ is the latent factors at time t and σ_*sigmoid*_(·) represents the sigmoidal activation function. The subscripts of weight matrix *W* indicate the corresponding gates that include forgetting gate *f*, input gate *i*, and output gate *o*. The information flow of the cell memory can be updated by

(21) $$\begin{array}{l} c_{u}=\;\sigma_{tanh}\left(W_{c,x}u_{t}+W_{c,1}h_{t-1}+b_{c}\right) \end{array}$$

(22) $$\begin{array}{l} c_{t}=f_{t}⊗c_{t-1}+i_{t}⊗c_{u} \end{array}$$

(23) $$\begin{array}{l} h_{t}=o_{t}⊗\sigma t_{tanh} \end{array}$$

where σ_*tanh*_ and ⊗ represent the hyperbolic tangent function and the element-wise product, respectively.

### Linear Kalman Filter

Linear Kalman filter is a commonly generative decoding method based on the linear dynamical. The hidden state *y*_*t*_ is a linear combination of the hidden state *y*_*t*−1_ (Wu et al., 2003). The system model can be described as

(24) $$\begin{array}{l} y_{t}=Ay_{t-1}+Q_{t-1} \end{array}$$

(25) $$\begin{array}{l} l_{t}=Hy_{t}+V_{t} \end{array}$$

where A denotes the system model parameter matrix, *H* is the observation model parameter matrix, and *Q* and *V* are the process noise and observation noise that obeys a Gaussian distribution. The observation vector *l*_*t*_ is the vector of latent factors. After converging the Kalman gain to its stable state early, we initialized *y*_0_ to the average of the observation vector at the beginning of every trial to predict *y*_*t*_.

## Results

### Some Specific Frequencies Are Dominant in the Digital Cognitive Process

**Figure 2:**
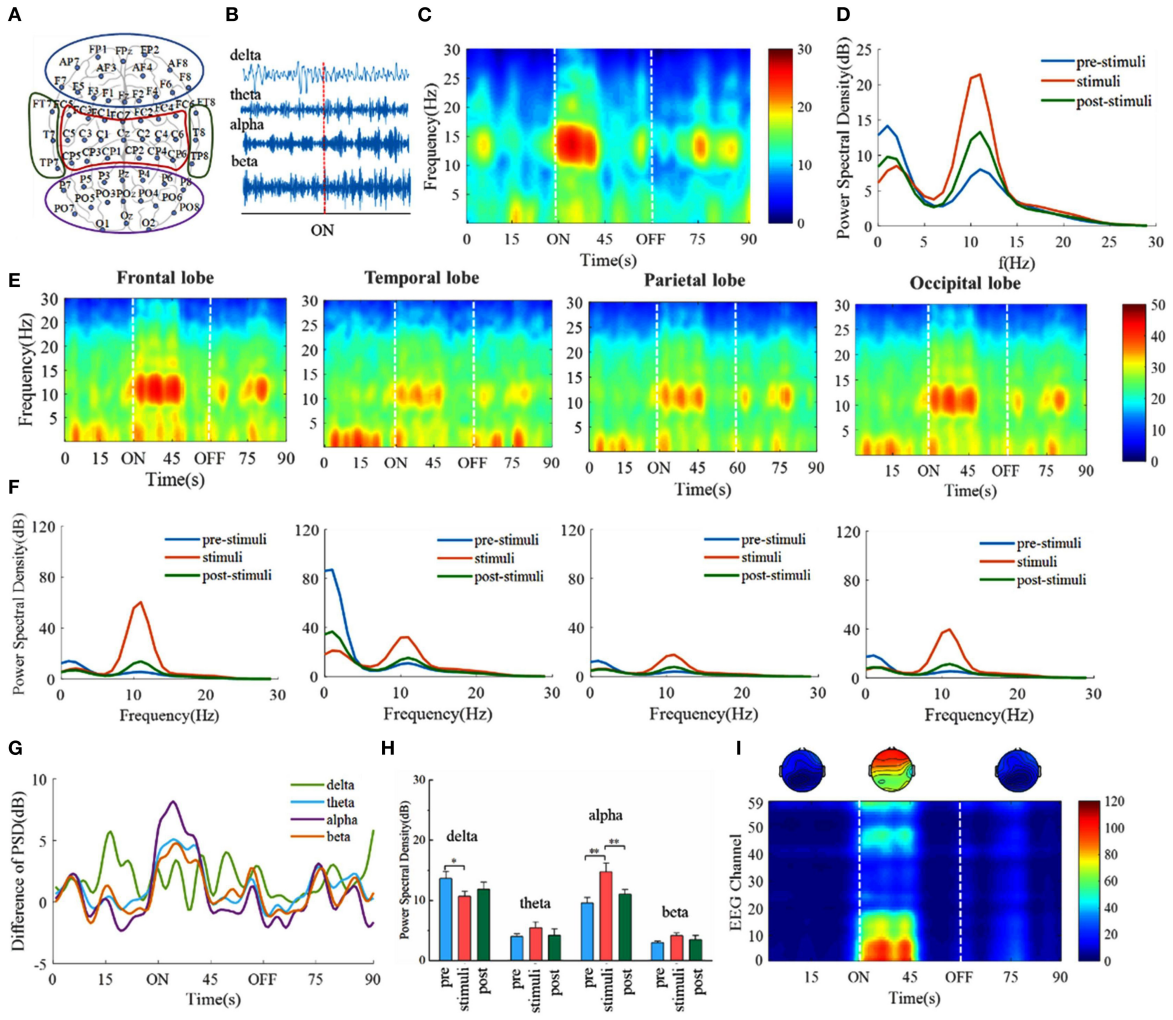
Take the number 1 as an example of visual stimuli, the frontal lobe alpha power saliently correlates with visual cognition. **(A)** Schematic drawing showing the location of EEG electrodes in the cerebral cortex. The schematic diagram of channel position with the frontal lobe is marked within blue lines and the temporal lobe, the parietal lobe, and the occipital lobe are marked with red, green, and purple lines, respectively. **(B)** Example EEG is divided into four subfrequency bands: delta (0.5–4 Hz), theta (4–8 Hz), alpha (8–15 Hz), beta (15–30 Hz). The red dotted line indicates the beginning of the visual stimulus. **(C)** EEG time-frequency spectrograms. 80-s time series raw EEG signals whereas the visual stimuli onset (30 s) and end (60 s) are marked with the white line. **(D)** Power spectrum density estimates for three states. **(E)** EEG time-frequency spectrograms of four lobes. **(F)** Power spectrum density of four lobes. **(G)** Change of averaged power across subjects in four frequency bands compared to the initial average power. **(H)** EEG power across four frequency bands. * *p* < 0.05, ** *p* < 0.01, one-way ANOVA followed by Bonferroni's *post hoc* test. Error bars describe standard error across subjects. **(I)** The variation of power of each EEG channel data in alpha.

We first investigate whether there exist specific frequencies that are dominant in the digital cognitive process. EEG recordings of the awake volunteers are performed to explore the changes in EEG evoked by digital cognition (Figure 2A). The EEG signals are divided into four major frequency bands involved in sensory consciousness, information coding, cognitive memory, selective attention: delta (0.5–4 Hz), theta (4–8 Hz), alpha (8–15 Hz), beta (15–30 Hz) (Figure 2B). In a dark behavioral chamber, presentations of visual stimuli (30 s) induce alpha frequency oscillations, but the oscillations persist only ~10 s during the digital stimuli period (Figures 2C,G). The frequency spectra show that the power of the alpha band increases during the stimuli period compared with that during the prestimuli and poststimuli periods (Figure 2D). Furthermore, the spectral power across four frequency bands is compared in Figures 2G,H. It is found that alpha band power increases during the stimuli period with *p* < 0.01 whereas delta band power decreases with *p* < 0.05 (one-way ANOVA followed by Bonferroni's *post-hoc* test).

### Specific Brain Areas Participate in the Visual Cognitive Task

The previous study has shown that specific brain areas may participate in specific cognitive functions (Gatti et al., 2020). The spectral power of the three periods across four brain lobes is calculated (Figures 2E,F). Alpha band power increases with the varying level in different brain lobes during the stimuli period, especially in the frontal and the occipital lobes. The variation of power of each EEG channel data in alpha is further analyzed and it is obtained that channels with relatively higher power spectrum values are mainly concentrated in the frontal and the occipital lobes (Figure 2I). In the following dimensionality reduction and decoding analysis, we are mainly focusing on the alpha frequency band and the first 10-s EEG signals during visual tasks. These results demonstrate that the alpha frequency band is the dominant frequency. Meanwhile, the frontal and the occipital lobes might be closely related to digital cognitive function.

### PCA Is Ineffective

**Figure 3:**
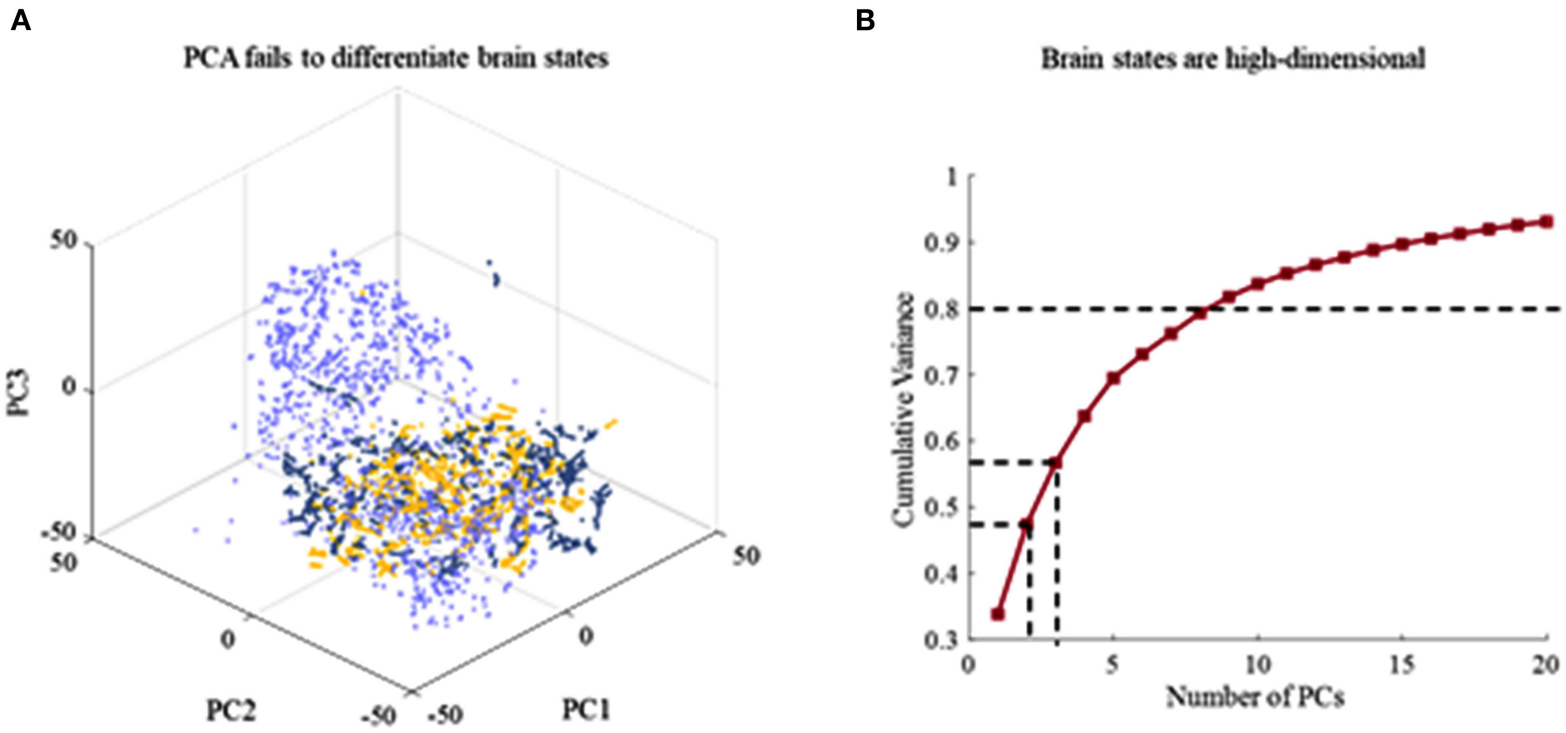
The result of dimensionality reduction using PCA. **(A)** Ineffective separation of brain dynamics at different task epochs by PCA. **(B)** Cumulative explained variance of brain states as a function of the number of PCs included. Explaining 80% of the total variance requires 9 dimensions.

To identify the dynamics of brain states of different cognitive processes, dimensionally reduction techniques that include PCA and t-SNE are further leveraged to estimate latent factors that reflect the subject's brain. Linear dimensionality reduction based on PCA is first performed. It is the ineffective separation of brain dynamics at different cognitive task epochs by PCA (Figure 3A). Three states are not possible to be distinguished by chosen viewing angle. It describes the cumulative explained variance of brain states as a function of the number of PCs in Figure 2B. It is obtained that the brain states are high-dimensional since explaining 80% of the total variance requires 9 dimensions at least. It is difficult to capture key differences during different task epochs using PCA, and thus, dimensionality reduction fails to achieve effectively in a fashion. To overcome this issue, a non-linear dimensionality reduction technique, t-distributed stochastic neighbor embedding (t-SNE), is applied to the time series of brain activities.

### Use t-SNE for Dimension Reduction

**Figure 4:**
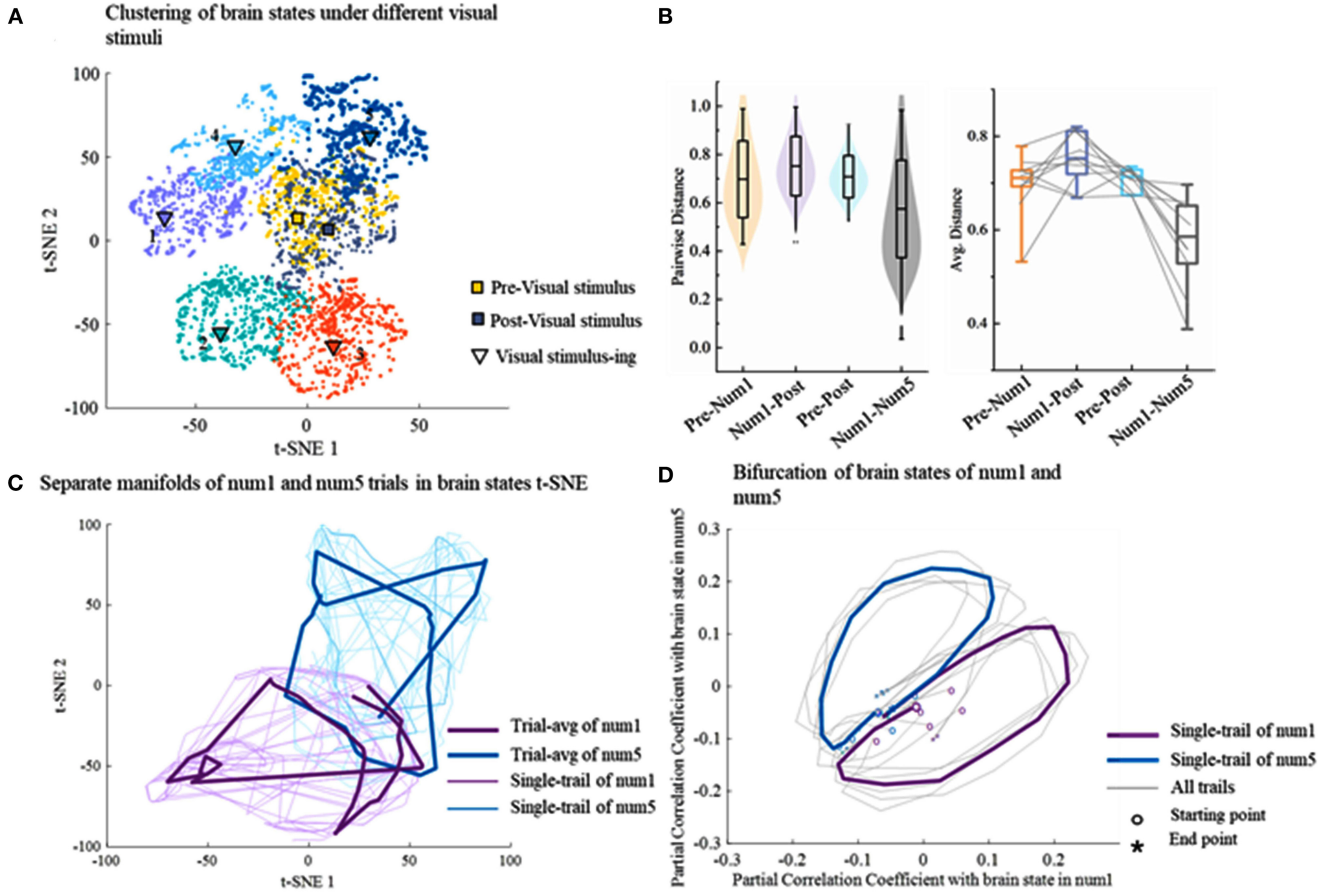
The result of dimensionality reduction using t-SNE. **(A)** The application of t-SNE on EEG data reveals seven distinct clusters corresponding to different tasks on a single trial. **(B)** The similarity of latent factor clusters within and across different tasks. The level of similarity is calculated by the pairwise Euclidean distances between brain states in the t-SNE space. The gray lines represent single trial. **(C)** Temporal evolution of brain states during different tasks lie on the separate cyclic manifold. The thin line represents a single trial and the thick line represents the trial average. **(D)** The bifurcation of brain states of different visual stimuli.

The non-linear dimensionality reduction technique t-SNE is used on the single trial, and the distributions of latent factors are shown in Figure 4A. Since the t-SNE is based on manifold learning, the latent factors after dimensionality reduction are divided into different manifolds. Except for a very small number of latent factors, the latent factors of the same task can be gathered in a region and the t-SNE clusters coincide well with distinct tasks. There are obvious classification boundaries among different tasks, which provides good conditions for the following decoding. More importantly, clusters in the rest states including prestimuli and poststimuli states are located in the same area. Latent factors from 10 experiments are further analyzed in the same way. The similarity of differences is measured *via* the pairwise Euclidean distances between brain states in the t-SNE space. The similarity between the clusters of two different tasks (Pre-Num1, Num1-Post, Num1–Num5) is lower than the similarity between the same tasks (pre–post) in Figure 4B. Different visual stimuli drive different transitions among brain states resulting in a lower similarity of the brain states between two different visual stimuli (Num1–Num5).

### Brain States Dynamically Evolve Into Cyclic Manifolds

To investigate the transition process of brain states, the consecutive brain states are temporally connected in the t-SNE map on the trial-by-trial level, which is shown in Figure 4C. It illustrates that the brain states dynamically evolve into cyclic manifolds which are distinct with different tasks over time, whereas the evolutive paths of brain states are highly similar during the same task. Afterward, to further analyze the difference among temporal evolutions of brain states during different cognitive tasks, the partial correlation coefficient is calculated between each brain state in a trial and a reference brain state determined by averaging all brain states of five tasks. The brain states are projected into 2D space spanned by reference brain states for the number of 1 and the number of 5. Strikingly, the bifurcation toward different cognitive tasks could be observed well at the beginning of visual stimuli at the single-trial level (Figure 4D), which further suggests that different visual cognitive tasks drive different transitions among brain states.

### Test Four Decoders Under PCA and t-SNE

**Figure 5:**
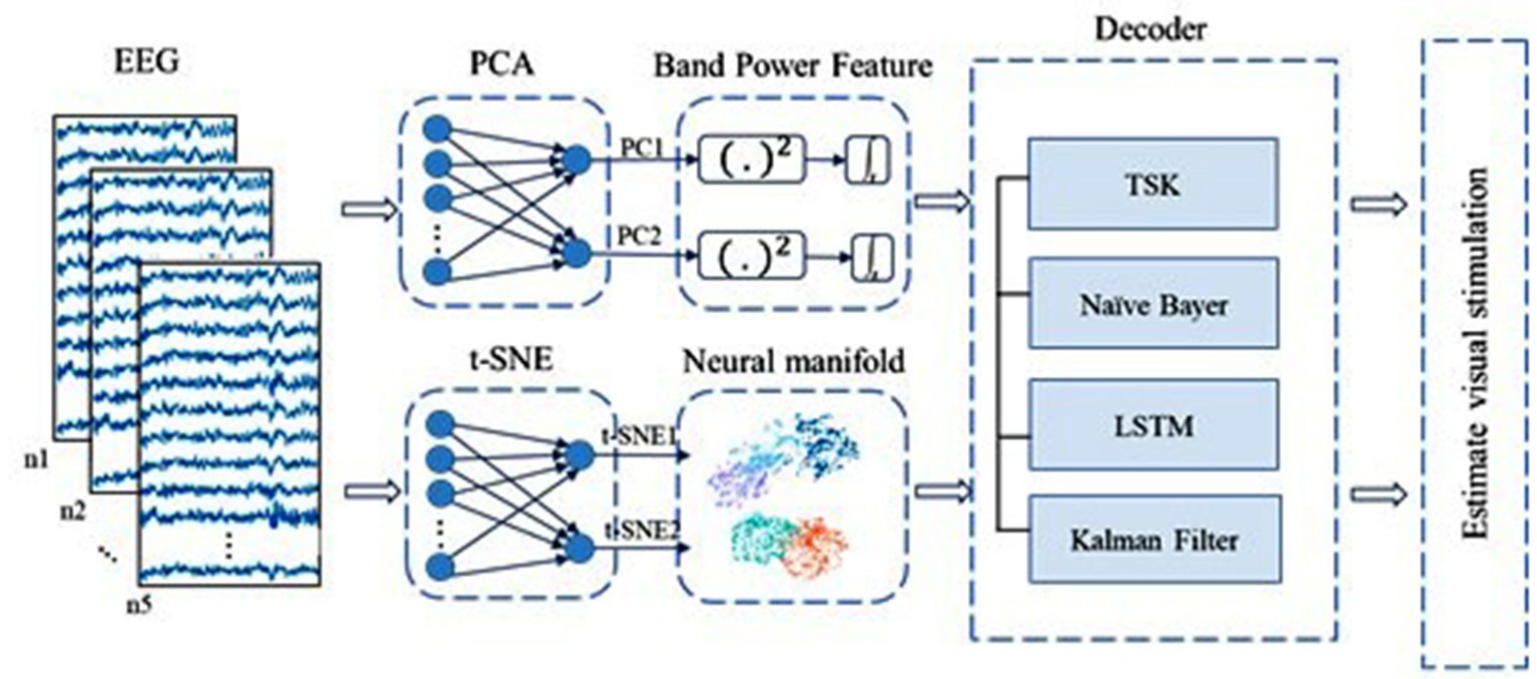
Overview of decoding based on two-dimensionality reduction techniques including PCA and t-SNE. EEG signals are down to two dimensions by PCA and t-SNE, respectively. For the two latent variables captured by PCA, we use their band power features in a short time as code to save their poor performance of original data. Meanwhile, latent variables from t-SNE are regarded as code directly. Decoders are TSK, NB, LSTM, and LKFS.

**Figure 6:**
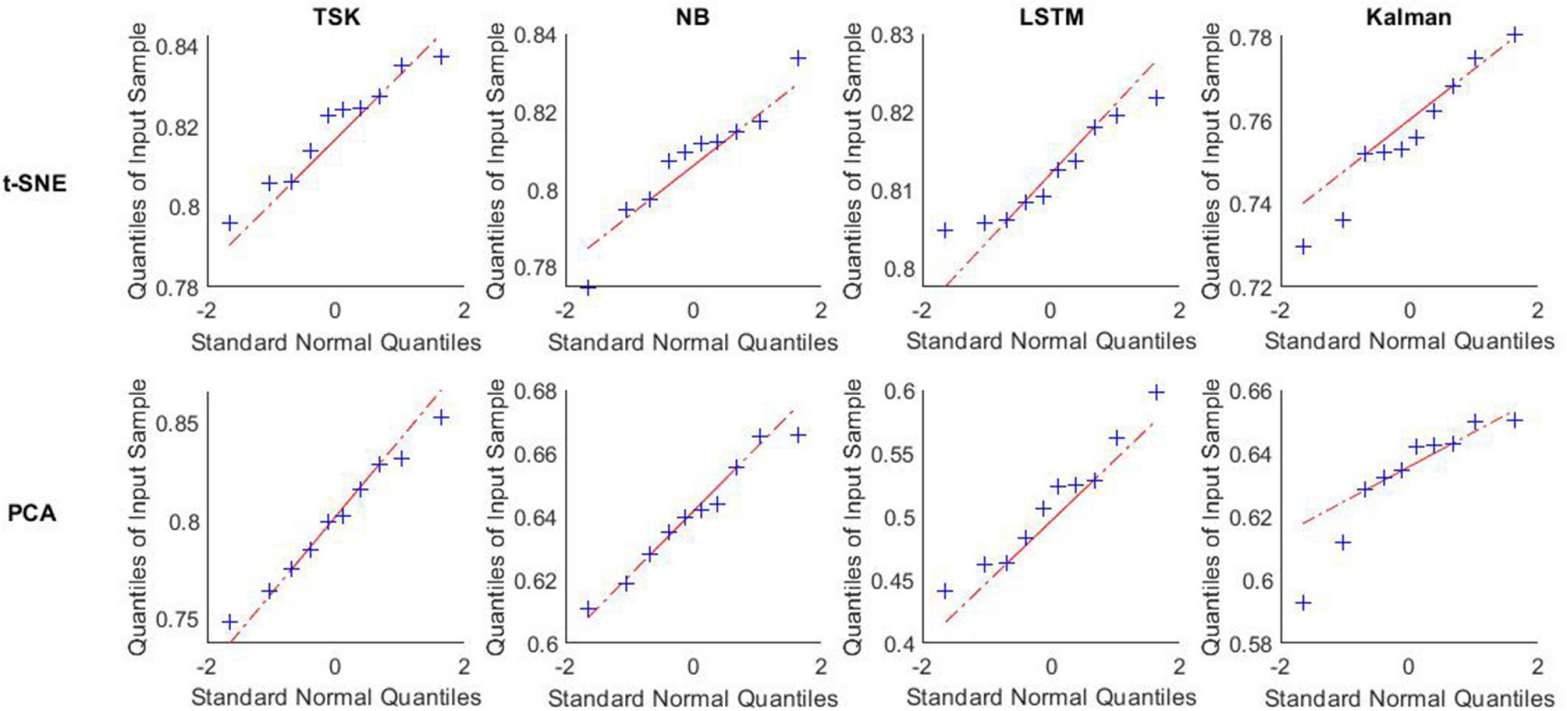
Q-Q plot of accuracy using different decoders after t-SNE and PCA vs. standard normal. Data about accuracy using different decoders after t-SNE and PCA in ten times trial are tested normality by q-q plot vs. standard normal. These decoders are TSK, NB, LSTM, and Kalman.

**Figure 7:**
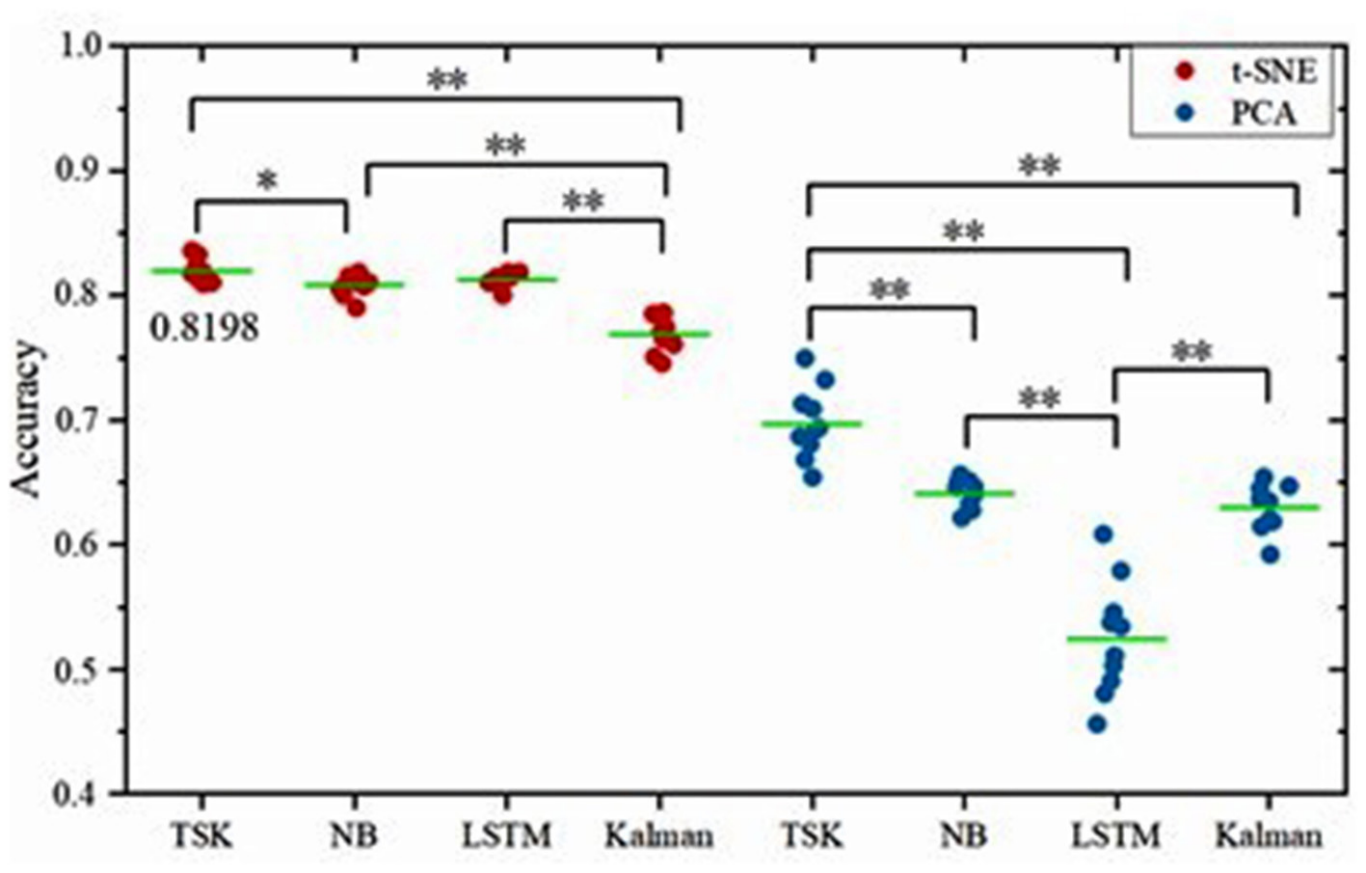
Comparison of the decoding accuracy for visual stimuli between two latent factors for each decoder. The green lines indicate the mean accuracy and the asterisks denote the significant degree of difference (*p < 0.05, **p < 0.01). The left column corresponds to the method of t-SNE and the right column corresponds to the method of PCA.

Given our analyses at the level of brain states and the latent factors under visual stimuli, it can be concluded that decoding brain activities by the non-linear dimensionality reduction technique is reliable. After dimension reduction, multiple machine learning decoders including linear decoder Kalman filter (KF), non-linear decoder long–short-term memory in recurrent neural networks (LSTM), fuzzy learning decoder Takagi-Sugeno-Kang fuzzy system (TSK), and naïve Bayes decoder (NB) are employed to decode brain activities. The decoding performance is evaluated for each combination of two-dimensionality reduction methods and four decoders (Figure 5). For the PCA method, the frequency band energy of the first two principal components is extracted, and the values *x*_1_, *x*_2_ are used as input vector x of the decoders. For the t-SNE method, the first two principal components *x*_1_, *x*_2_ are retained directly to obtain the neural manifold, which is used as the input vector x to the decoders. The decoding is implemented through 10-fold crossvalidation, which means that 9 out of 10 samples are selected as a training set, and the remaining 1 out of 10 samples are selected as the test set in each classification process. The classification process is repeated ten times, and the average value of the 10 times is taken as the final result. TSK decoder displays the encouraging consequence with 0.8198 accuracies with t-SNE and 0.6971 accuracies with PCA. The features extracted by the non-linear manifold learning method have better decoding performance than that of PCA. The performance of TSK is compared with three common methods (NB, LSTM, LKF) for EEG decoding. The accuracy data are normally distributed as the results of the Q-Q plot are approximately on a straight line (Figure 6). The classification results are shown in Table 1, Figure 7. Table 1 shows the classification accuracy of different decoders with two whole-brain features extracted by PCA and t-SNE. Meanwhile, TSK can achieve high performance in decoding EEG. The comparison of the decoding accuracy for visual stimuli across different latent factors and decoders is depicted in Figure 7. Friedman's test with Bonferroni correction is used to analyze the difference between the decoding accuracy of four decoders. The Friedman's test reveals the main effect of decoders on the decoder accuracy when using t-SNE (χ^2^ = 20.97, *p* < 0.01) or when using PCA (χ^2^ = 25.51, *p* < 0.01). When using t-SNE, the linear decoder KF shows lower accuracy than other decoders (*p* < 0.01) and TSK shows higher accuracy than NB (*p* < 0.05), whereas there is no difference between TSK and NB, LSTM. When using PCA, the TSK shows higher accuracy than other decoders (*p* < 0.01), and NB and KF also show higher accuracy than LSTM (*p* < 0.01). These indicate that TSK has a good decoding effect in both linear and non-linear dimensionality reduction techniques. Meanwhile, for all decoders, the t-SNE dimensionality reduction technique has a better decoding effect than PCA.

**Table 1:** Decoding accuracy of different decoders with two whole-brain features extracted by PCA and t-SNE.

**Method**	**TSK**	**NB**	**LSTM**	**Kalman**
t-SNE	0.8198 ± 0.0088	0.8081 ± 0.00820	0.8124 ± 0.0052	0.7692 ± 0.0143
PCA	0.6971 ± 0.0287	0.6412 ± 0.0112	0.5241 ± 0.0460	0.6293 ± 0.0186

### TSK Rules

**Figure 8:**
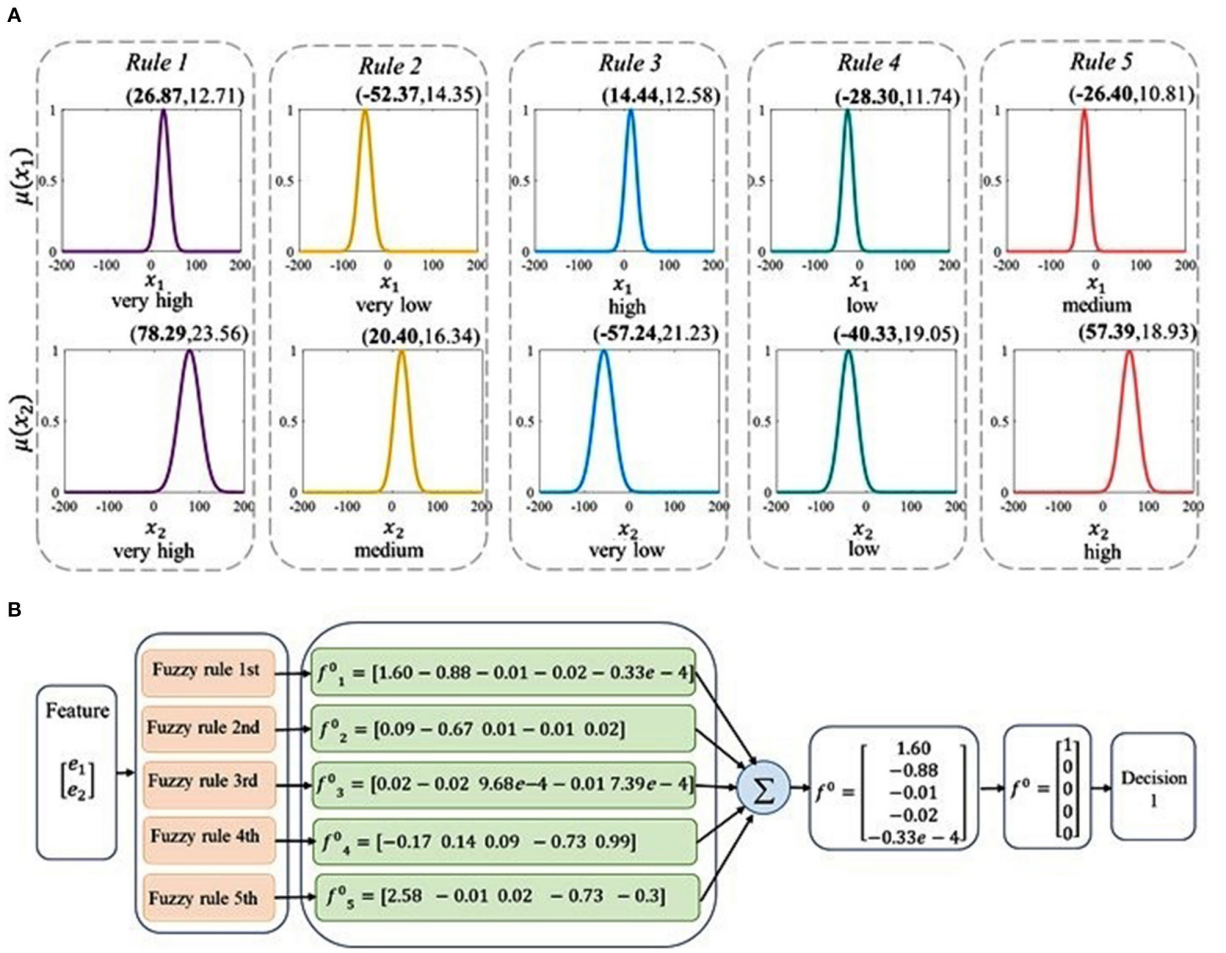
The decoding principle of the TSK method. **(A)** The descriptions of the antecedent linguistic and membership functions of five rules in the TSK decoder. In each subgraph, the horizontal coordinate and the vertical coordinate denote the independent inputs and the membership functions, respectively. The antecedent parameters (*centers, standard variance*) are presented above the corresponding membership function curve. Fuzzy linguistic descriptions are shown below the membership functions. **(B)** The identification process of visual information *via* five fuzzy rules and the fuzzy decoder.

The brain activities are first decoded using the TSK decoder. The example of TSK with five rules is illustrated in Figure 8. It illustrates the corresponding membership functions of each fuzzy set obtained from five fuzzy rules in Figure 8A. Each membership function corresponds to a fuzzy linguistic description which is *very high, high, medium, low, and very low*. To provide further explanation, the antecedent parameters that include centers and standard variance are calculated, which are presented in Figure 8A. By the permutation of five linguistic descriptions, there can be twenty-five rules. Take five of them as an example. Based on the linguistic expressions and the corresponding linear function, the fuzzy rules can be shown as follows:

*Rule 1*: IF *x*_1_ is *very high* AND *x*_2_ is *very high*,

(26) $$\begin{array}{l} \text{THEN}\\ f_{1}=\left[\begin{array}{c} 1.8268-0.0113x_{1}-0.0042x_{2},\\ 0.2707-0.0144x_{1}-0.0119x_{2},\\ -0.2911-0.0038x_{1}-0.0052x_{2},\\ 0.0370+7.2537e-4x_{1}+9.4636e-4x_{2},\\ -0.0289-8.1321e-4x_{1}-0.0010x_{2} \end{array}\right] \end{array}$$

*Rule 2*: IF *x*_1_ is *very low* AND *x*_2_ is *medium*,

(27) $$\begin{array}{l} \text{THEN}\\ f_{2}=\left[\begin{array}{c} -0.0954-4.2010e-4x_{1}-0.0013x_{2},\\ -0.0146-6.0436e-4x_{1}-8.2373e-5x_{2},\\ 0.1950+0.0020x_{1}-0.0026x_{2},\\ 1.5912-0.0135x_{1}+5.2341e-4x_{2},\\ -0.8781-0.0111x_{1}-0.0034x_{2} \end{array}\right] \end{array}$$

*Rule 3*: IF *x*_1_ is *high* AND *x*_2_ is *very low*,

(28) $$\begin{array}{l} \text{THEN}\\ f_{3}=\left[\begin{array}{c} -0.5874-0.0093x_{1}-0.0024x_{2},\\ 1.7819-0.0032x_{1}-0.0021x_{2},\\ -1.0105-0.0120x_{1}+8.3280e-4x_{2},\\ 0.0375-8.7808e-4x_{1}-2.2777e-5x_{2},\\ 0.0814-0.0023x_{1}+0.0027x_{2} \end{array}\right] \end{array}$$

*Rule 4*: IF *x*_1_ is *low* AND *x*_2_ is *low*,

(29) $$\begin{array}{l} \text{THEN}\\ f_{4}=\left[\begin{array}{c} -0.1187+0.0017x_{1}+5.0977e-4x_{2},\\ -1.1958+0.0088x_{1}+0.0153x_{2},\\ 3.1966+0.0236x_{1}-0.0070x_{2},\\ 0.0791+0.0020x_{1}+0.0027x_{2},\\ -0.6716+0.0071x_{1}-0.0145x_{2} \end{array}\right] \end{array}$$

*Rule 5*: IF *x*_1_ is *medium* AND *x*_2_ is *high*,

(30) $$\begin{array}{l} \text{THEN}\\ f_{5}=\left[\begin{array}{c} 0.1576+0.0030x_{1}-0.0014x_{2},\\ -1.0899-0.0099x_{1}+0.0139x_{2},\\ -0.7449+0.0117x_{1}-0.0041x_{2},\\ 0.0255+6.8180e-4x_{1}-1.8482e-5x_{2},\\ 2.4971+0.0055x_{1}+0.0163x_{2} \end{array}\right] \end{array}$$

Based on these five fuzzy rules above, the mechanism of the decoding process is depicted in Figure 8B. The low-dimensional characteristics of different tasks are taken as the inputs of the decoder, and the label vector is predicted by the TSK system's decision output. The sum of the decision outputs ${f^{0}}_{i}\left(i=1,2,\ldots ,5\right)$ based on the five rules is indicated by *f*^0^ and the decision outputs are handled by setting the maximal element in *f*^0^ to 1 and others to 0. In this example, the decision output is number 1.

### Brain Activities Are Decoded With the Combination of Data of Different Brain Regions

**Figure 9:**
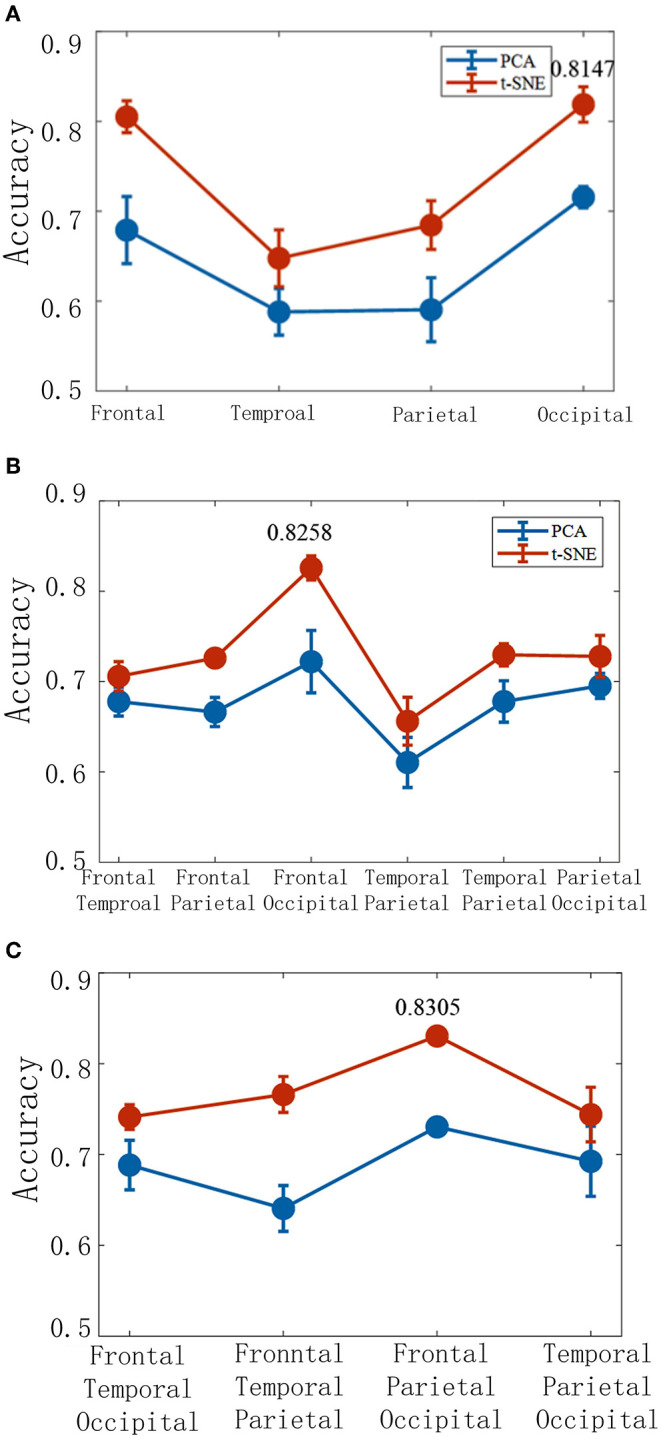
The corresponding decoding results under different brain lobe associations. The accuracy of the TSK decoder is based on two latent factors with a single brain lobe **(A)**, two brain lobes **(B)**, and three brain lobes **(C)**.

The brain activities are decoded with the low-dimensional features extracted from a single brain lobe, two brain lobes, and three brain lobes (Figure 9). The result shows that the frontal and the occipital lobes are the top two highest accuracies when the features are extracted from a single brain lobe. The combination of the frontal and the occipital lobes is the highest accuracy in the two lobes combination. Additionally, in the three lobes combination, the frontal and occipital and parietal lobes are the highest effective. We hence infer that the frontal and the occipital lobes with high accuracy may be the key areas of the brain's visual cognition process. It can be concluded that finding the dominant brain regions can improve decoding efficiency and accuracy, which is essential for BCI.

## Discussions

### Use Neural Manifold to Decode Cortical Activity Under Visual Stimuli

In this work, we propose a method to decode cortical activity under visual stimuli from a single-trial level using the neural manifold. Through power spectrum analysis, it is found that visual cognition induces significant changes in the alpha band (Hogendoorn and Burkitt, 2018). Different visual stimuli lead to different spiking of neurons, which represent different information encoded by the human brain. The information is recorded in EEG and is exposed as the bifurcated manifold by dimension reduction methods. By further analyzing the latent factors extracted from the alpha band and using it to decode the brain activities, it is found that five major clusters that are associated with the visual stimuli distinctively occupy separate subregions.

### t-SNE Has a Better Performance

The dimensionality reduction techniques are compared in terms of decoding performance. As excepted, it is confirmed that the latent factors obtained by t-SNE well-decoded brain activities. Decoding visual information using latent factors estimated by the t-SNE results in a superior performance to another case using PCA-estimated latent factors regardless of the decoder types, that is, TSK, NB, LSTM, and LKF. The research on the neurodynamic of the cerebral cortex has consistently uncovered low-dimensional manifolds that capture a crucial portion of neural variability (Arieli et al., 1996). PCA is widely used in motor cortex dynamics research. Gallego et al. (2018) confirmed the existence of a neural manifold in the motor cortex of monkeys by PCA. However, for behaviors whose dynamics explore non-motor brain areas, estimating neural manifolds using linear techniques requires latent factors of higher dimensions than the true dimensions in the data. EEG is commonly considered to have significant chaotic characteristics, linear methods provided poor estimates of the neural manifold (Figure 3). When brain activity is reduced to the same dimensions with xx, we can obtain a better recognition performance on the latent factors decoded by t-SNE during different cognitive tasks (Figure 4). The improvement in decoding brain activities using t-SNE suggests that the brain activities lie on a low-dimensional, non-linear manifold in the high-dimensional space and t-SNE can truly model the hidden state space of the brain in the cognitive process.

### TSK Has the Best Performance Among the Four Decoders

This may be due to TSK's good non-linear approximation ability as the performance of decoders is significantly more improved using latent factors estimated by t-SNE than PCA. Additionally, TSK could transform the decision output into a value between 0 and 1 through fuzzy rules, which is closer to human thoughts than other dichotomies of either 0 or 1 (Yu et al., 2019a). These findings suggest that we can design a simple fuzzy learning decoder (TSK) with t-SNE while achieving well performance. Multiple supervised machine learning methods that include TSK, NB, LSTM, KF are employed to classify different cognitive tasks based on two-dimensionality reduction techniques. Using latent factors estimated by t-SNE to decode visual information has higher accuracy than those estimated by PCA. Among these decoders, TSK with a promising classification result has been proved to be useful in the decoding analysis of neuroimaging data in many applications (Yu et al., 2019b).

### The Frontal and the Occipital Lobes Are Found to Be the Most Effective Areas

According to the classification results, it is obtained that the number of brain lobes is not proportional to the classification performance (Figure 7). The frontal and the occipital lobes are found to be the most effective areas to classify different cognitive tasks. Compared with traditional methods, the visual decoding approach proposed has lower implementation difficulty and higher performance. Future work will consider finding key channels and frequencies to further reduce the amount of data analysis and improve decoding accuracy. The dominant brain lobes are found based on the decoding accuracy, but how to select features more efficiently and accurately is necessary to be further considered. In future works, it will be combined common spatial patterns (CSPs) with deep learning theory, further ensuring optimal channels and latent factors that are closely related to cognitive processes to achieve higher decoding accuracy.

## Conclusion

In this work, a novel decoding model combining manifold dimensionality reduction approaches with machine learning is designed. By only capturing EEG signals from key brain regions, researchers can obtain the same or better decoding performance. Meanwhile, the overall computational efficiency of the visual information decoding can be improved easily with the reduction of the originally collected datasets. In particular, the TSK method integrates the advantages of fuzzy rules and membership function, so the TSK method has higher interpretability and robustness (Takagi and Sugeno, 1985; Azeem et al., 2000; Kuncheva, 2000). The work provides effective algorithms for the accurate control of BCI and is of great significance to the rehabilitation training and treatment of brain-related neurological diseases.

## Data Availability Statement

The raw data supporting the conclusions of this article will be made available by the authors, without undue reservation.

## Ethics Statement

Ethical review and approval was not required for the study on human participants in accordance with the local legislation and institutional requirements. The patients/participants provided their written informed consent to participate in this study.

## Author Contributions

JW and HY designed the study. QZ, SL, and KL performed the research. QZ wrote the manuscript.

## Funding

This work was supported in part by the National Natural Science Foundation of China under Grant 62173241, in part by the Natural Science Foundation of Tianjin, China (Grant Nos. 19JCYBJC18800 and 20JCQNJC01160), the Foundation of Tianjin University under Grant 2020XRG-0018, and Tianjin Research Innovation Project for Postgraduate Students (Grant No. 2021YJSB188). The authors also gratefully acknowledge the financial support provided by Opening Fundation of Key Laboratory of Opto-technology and Intelligent Control (Lanzhou Jiaotong University), Ministry of Education (KFKT2020-01).

## Conflict of Interest

The authors declare that the research was conducted in the absence of any commercial or financial relationships that could be construed as a potential conflict of interest.

## Publisher's Note

All claims expressed in this article are solely those of the authors and do not necessarily represent those of their affiliated organizations, or those of the publisher, the editors and the reviewers. Any product that may be evaluated in this article, or claim that may be made by its manufacturer, is not guaranteed or endorsed by the publisher.
